# Prostaglandin (PG) F2 Alpha Synthesis in Human Subcutaneous and Omental Adipose Tissue: Modulation by Inflammatory Cytokines and Role of the Human Aldose Reductase AKR1B1

**DOI:** 10.1371/journal.pone.0090861

**Published:** 2014-03-24

**Authors:** Andréanne Michaud, Nicolas Lacroix-Pépin, Mélissa Pelletier, Alain Veilleux, Suzanne Noël, Céline Bouchard, Picard Marceau, Michel A. Fortier, André Tchernof

**Affiliations:** 1 Endocrinology and Nephrology, Laval University Medical Center, Quebec City, Canada; 2 Department of Nutrition, Laval University, Quebec City, Canada; 3 Gynecology Unit, Laval University Medical Center, Quebec City, Canada; 4 Reproduction and Biology, Laval University Medical Center, Quebec City, Canada; 5 Department of Surgery, Quebec Cardiology and Pulmonology Institute, Quebec City, Canada; Fudan University, China

## Abstract

**Introduction:**

PGF_2α_ may be involved in the regulation of adipose tissue function.

**Objectives:**

1) To examine PGF_2α_ release by primary preadipocytes, mature adipocytes and whole tissue explants from the subcutaneous and omental fat compartments; 2) To assess which PGF synthase is the most relevant in human adipose tissue.

**Methods:**

Fat samples were obtained by surgery in women. PGF_2α_ release by preadipocytes, adipocytes and explants under stimulation by TNF-α, IL-1β or both was measured. Messenger RNA expression levels of *AKR1B1* and *AKR1C3* were measured by RT-PCR in whole adipose tissue and cytokine-treated preadipocytes. The effect of AKR1B1 inhibitor ponalrestat on PGF_2α_ synthesis was investigated.

**Results:**

PGF_2α_ release was significantly induced in response to cytokines compared to control in omental (p = 0.01) and to a lesser extent in subcutaneous preadipocytes (p = 0.02). Messenger RNA of *COX-2* was significantly higher in omental compared to subcutaneous preadipocytes in response to combined TNF-α and IL-1β (p = 0.01). Inflammatory cytokines increased AKR1B1 mRNA expression and protein levels (p≤0.05), but failed to increase expression levels of *AKR1C3* in cultured preadipocytes. Accordingly, ponalrestat blunted PGF_2α_ synthesis by preadipocytes in basal and stimulated conditions (p≤0.05). Women with the highest PGF_2α_ release by omental adipocytes had a higher BMI (p = 0.05), waist circumference (p≤0.05) and HOMAir index (p≤0.005) as well as higher mRNA expression of *AKR1B1* in omental (p<0.10) and subcutaneous (p≤0.05) adipose tissue compared to women with low omental adipocytes PGF_2α_ release. Positive correlations were observed between mRNA expression of AKR1B1 in both compartments and BMI, waist circumference as well as HOMAir index (p≤0.05 for all).

**Conclusion:**

PGF_2α_ release by omental mature adipocytes is increased in abdominally obese women. Moreover, COX-2 expression and PGF_2α_ release is particularly responsive to inflammatory stimulation in omental preadipocytes. Yet, blockade of PGF synthase AKR1B1 inhibits most of the PGF_2α_ release.

## Introduction

Expansion of body fat mass as seen in obesity is related to alterations of the metabolic and endocrine function of adipose tissue leading to poor handling of postprandial lipids, fatty acid spillover to other tissues and organs, macrophage infiltration and release of pro-inflammatory mediators [1]–[5]. This condition has been proposed as a potential mechanism linking obesity with metabolic diseases [4]–[6]. Among the many inflammatory factors secreted by adipose tissue, prostaglandins (PGs) have been proposed as contributing mediators of inflammation in obesity, hyperinsulinemia, hypertension and cardiovascular disease [7]–[9].

Whole tissue explants, isolated mature adipocytes and non-fat cells from the stromal-vascular fraction were used by other groups to study PG release or synthesis by the various cell fractions of adipose tissue [10]–[20]. Taken together, these studies indicate that mature adipocytes and stromal-vascular cells both contribute to the synthesis and release of PGs, the main secreting cells being the non-fat, stromal-vascular fraction of adipose tissue [10], [11], [15], [21]. Mature adipocytes from humans, mice or rats are also known to release PGs including PGE_2_ and PGF_2α_ as well as PGI_2_ and other metabolites [10], [13], [15]. Recent *in vitro* studies demonstrated that PGD_2_ and PGI_2_ enhance adipocyte differentiation [22], [23], while others have shown that PGE_2_ and PGF_2α_ inhibit this process through their specific receptors, the EP4 receptor [24] and the FP receptor respectively [25]–[28]. These findings suggest that PGF_2α_ may have anti-adipogenic functions.

PGs are derived from arachidonic acid (AA) through the activity of two PGH synthases (PTGS), the constitutive cyclooxygenase (COX)-1 or the inducible COX-2, both converting AA consecutively into PGG_2_ and PGH_2_ [29]. PGF_2α_ is mostly synthesized by the reduction of the 9,11-endoperoxide moiety of PGH_2_ [30]. PGF_2α_ may also be formed through reduction of PGD_2_ by 11-keto reductase or PGE_2_ by 9-ketoreductase [31]. These reactions are catalyzed by enzymes of the aldo-keto reductase (AKRs) family [31], which are monomeric, soluble oxido-reductases dependent on NAD(P)H. In mammals, the first PGF synthase identified belongs to the AKR1C family (AKR1C3) [32]. We also demonstrated in other models including bovine and human endometrium or other tissues that enzymes of the AKR1B family exhibit PGF synthase activity [33]–[35]. *In vitro* studies have previously confirmed the PGF synthase activity of mouse Akr1b3 and Akr1b7 as well as human AKR1B1 [36]. A recent study also demonstrated that Akr1b3, the murine ortholog of human AKR1B1, acts as a functional PGF synthase and is involved in the suppression of adipogenesis through the FP receptor in the 3T3-L1 cell line [37]. The relevance of these data in humans remains to be established.

Considering that human body fat distribution is quite heterogeneous and that excess abdominal adipose tissue accumulation is associated with increased cardiometabolic risk independent of total body fat mass [38], depot-specific differences in PGF_2α_ synthesis may have a pathophysiological role in the development of visceral obesity-related comorbidities. Until now, whether and how PGF_2α_ release by mature adipocytes from the subcutaneous and omental fat depot is affected in human obesity has never been clearly established. The aim of this study was to examine PGF_2α_ release by primary preadipocytes, mature adipocytes and whole tissue explants from the subcutaneous and visceral (omental) fat compartments. We tested the hypothesis that preadipocytes from the omental fat compartment release more PGF_2α_ than those from the subcutaneous depot and that abdominal obesity is related to alterations in PGF_2α_ release by the mature cell fraction from the omental fat compartment. Since previous literature did not allow determining which PGF synthase is the most relevant in human adipose tissue, we also focused on AKR1B1 and AKR1C3 in response to inflammatory cytokines in human preadipocyte cultures, and investigated the impact of an AKR1B1 inhibitor on PGF_2α_ synthesis. We tested the hypothesis that AKR1B1 inhibition significantly impairs the synthesis of PGF_2α_ by human cultured preadipocytes.

## Materials and Methods

### Subjects and ethics statement

The study sample included lean to obese women recruited through the elective surgery schedule of the Gynecology Unit at Laval University Medical Center. Women were undergoing gynecological surgery for total or subtotal abdominal hysterectomies. The study was approved by the Research Ethics Committees of Laval University Medical Center (protocol C09-08-086). We also included cultures from women undergoing biliopancreatic diversion for the treatment of morbid obesity (n = 2, aged 42 and 53 years, BMI 40 and 52.7 kg/m^2^) with approval from the Research Ethics Committees of the Quebec Cardiology and Pulmonology Institute (protocol CERHL 1142). All subjects provided written informed consent before their inclusion in the study.

### Clinical parameters and glucose homeostasis measurements

Body weight, height, body mass index (BMI) and waist circumference were measured on the morning of the surgery according to a standard protocol. Fasting glucose and insulin were measured in pre-surgery blood samples collected after a 12 h-overnight fast. Plasma glucose was measured using the glucose oxidase method and plasma insulin levels were measured by ELISA (Millipore, St.Charles, MO, USA). The HOMA insulin resistance index (HOMA_ir_) was calculated as described [39].

### Adipose tissue sampling

Subcutaneous and omental adipose tissue samples were respectively collected at the site of the surgical incision and greater omentum and immediately carried to the laboratory. A portion of the fresh sample was used for adipocyte and preadipocyte isolation and pieces of fresh adipose tissue samples were fixed in 10% formalin for 24–48 hours at room temperature prior processing for routine paraffin wax embedding. A proportion of 30 mg fresh adipose tissue was cut into 5–10 mg pieces and placed in serum-free Medium 199. Adipose tissue explants were kept in culture at 37°C under a 5% CO_2_ atmosphere. The remaining portion of the sample was immediately frozen and kept for future analyses.

### Adipocyte isolation and adipocyte size measurement

A portion of each fresh tissue sample was digested 45 min at 37°C with type I collagenase in Krebs-Ringer-Henseleit (KRH) buffer supplemented with glucose, adenosine, ascorbic acid and BSA according to a modified version of the Rodbell method [40]. Digested tissues were filtered through nylon mesh and mature adipocytes were separated from the stromal-vascular fraction by floatation. Cells were washed 3 times and mature adipocyte suspensions were visualized using a phase contrast microscope attached to a camera and computer interface. Pictures of the suspensions were taken and the Scion Image software was used to measure the size (diameter) of 250 adipocytes for each tissue sample. Average adipocyte size of each sample was used in analyses.

### Preadipocyte isolation and primary cultures

Preadipocytes were isolated using a modification of the Van Harmelen method [41]. The residual KRH buffer of the adipocyte isolation, which contained the stromal-vascular fraction, was centrifuged and the pellet was washed in DMEM-F12 culture medium supplemented with 10% calf serum, 2.5 μg/ml amphotericin B and 50 μg/ml gentamicin. Stromal-vascular cells were then filtered through 140 μm nylon mesh to remove endothelial/mesothelial cells, placed in culture plates and cultured at 37 °C under a 5% CO_2_ atmosphere. Medium was changed every 2–3 days.

### PGF_2α_ measurements

PGF_2α_ release by isolated subcutaneous and omental mature adipocytes was measured in suspensions of approximately 5000 cells incubated for 2 h at 37 °C in KRH buffer. The PGF_2α_ response of mature adipocytes to inflammatory cytokine stimulation was tested by incubating suspensions with TNF-α (1 ng/ml) and/or IL-1β (1 ng/ml) or vehicle for 2 hours. The PGF_2α_ response of primary preadipocytes and primary organ cultures to inflammatory cytokines was assessed by incubating the cells/explants with TNF-α (1 ng/ml) and/or IL-1β (1 ng/ml) or vehicle for 24 hours. The incubation time with inflammatory stimuli was established according to a time-course experiment (0, 3 h, 6 h, 16 h and 24 h) and the dose of inflammatory cytokines was established according to a dose-response experiment (0.01 ng/ml, 0.1 ng/ml, 1 ng/ml, 10 ng/ml and 100 ng/ml). Taking into consideration that mature fat cells cannot be kept more than a few hours in suspension, a short incubation (2 hours) was performed with this fraction. The PGF_2α_ response of primary preadipocytes was also assessed by incubating cells with IL-1β (1 ng/ml) or vehicle in the presence or absence of the aldose reductase inhibitor ponalrestat (0.05, 0.5, 5, 10 or 20 μM) [42] for 24 hours. Aldose reductase inhibitor ponalrestat was from Tocris Bioscience (Ellisville, MO, USA). Cytotoxicity was assessed by the measurement of adenylate kinase release in the medium using ToxiLight Non-destructive cyclooxygenase bioassay kit (Lonza, Rockland, ME, USA). PGF_2α_ content in the media was measured by enzyme immunoassay, and acetylcholinesterase-linked PGF_2α_ tracer (Cayman) as previously described [43]. Considering the nature and cultivability of each cell type, PGF_2α_ release by omental and subcutaneous mature adipocytes was expressed as pg/10^6^ cells*2 h. PGF_2α_ release by cultured primary subcutaneous and omental preadipocytes was expressed as pg/ml*μg protein*24 h and PGF_2α_ release by omental and subcutaneous adipose tissue explants was expressed as pg/ml*mg tissue*24 h. Recombinant human TNF-α and IL-1β were purchased from PeproTech (Rocky Hill, NJ, USA).

### Messenger RNA expression by quantitative real-time RT-PCR

Total RNA was extracted using the RNeasy lipid tissue extraction kit and on-column DNase treatment (Qiagen, Valencia, CA, USA) from whole subcutaneous and omental adipose tissue or from preadipocyte cultures treated with TNF-α and/or IL-1β or vehicle. RNA quality and concentration was assessed using the Agilent Technologies 2100 bioanalyzer (Agilent, Santa Clara, CA, USA). Complementary DNA was generated from total RNA using random hexamers, oligo dT_18_ and Superscript III Rnase H-RT (Invitrogen Life Technologies, Burlington, ON, Canada) and purified with QIAquick PCR Purification Kit (Qiagen, Valencia, CA, USA). Real-time cDNA amplification was performed in duplicate using the LightCycler 480 (Roche Diagnostics, Mannheim, DE, USA) and the SYBR Green I Master (Roche Diagnostics, Indianapolis, IN, USA). The conditions for PCR reactions were: 45 cycles, denaturation at 95°C for 10 sec, annealing at 60°C for 10 sec, elongation at 72°C for 14 sec and then 74°C for 5 sec (reading). A melting curve was generated to assess non-specific signal. Calculation of the number of copies of each mRNA was performed according to Luu-The et al. [44] using second derivative method and a standard curve of Cp versus logarithm of the quantity. The standard curve was established using known amounts of purified PCR products and the LightCycler 480 v1.5 program provided by the manufacturer (Roche Diagnostics, Mannheim, DE, USA). PCR amplification efficiency was verified. Target gene amplifications were normalized using housekeeping gene expression levels of ATP synthase O subunit (*ATP5O*) for whole tissue extracts or Glucose-6-phosphate dehydrogenase (*G6PD*) for stimulated preadipocytes. Expression levels of *ATP5O* were not different in omental versus subcutaneous adipose tissue and were not associated with adiposity measurements in our study sample. *G6PD* mRNA expression was not significantly modulated during inflammatory cytokine stimulation in preadipocytes. The transcripts examined were *COX-2* and the two putative PGF synthases, *AKR1B1* and *AKR1C3*. Primer sequences were designed using GeneTools (Biotools Inc., Jupiter, FL, USA) and are listed in **Table 1**. Quantitative realtime PCR measurements were performed by the CHU de Québec Research Center Gene Expression Platform (Quebec, Canada).

**Table 1:** **Oligonucleotides used in real-time RT-PCR quantification.**

Gene Symbol	Description	GenBank	Oligonucleotide Sequence 5'→ 3' Forward/Reverse
***AKR1B1***	Aldo-keto reductase family 1B1	NM_001628	GATCGCAGCCAAGCACAATAA/ACAGCTCAACAAGGCACAGAC
***AKR1C3***	Aldo-keto reductase family 1C3	NM_003739	CAACCAGGTAGAATGTCATCCGTAT/ACCCATCGTTTGTCTCGTTGA
***COX-2***	Cyclooxygenase 2	NM_000963	ATGGGTAATGTTATATGTTCTCCTGC/TGGTGACTGTTTTAATGAGCTCTG
***ATP5O***	ATP synthase O subunit	NM_001697	ATTGAAGGTCGCTATGCCACAG/AACGACTCCTTGGGTATTGCTTAA
***G6PD***	Glucose-6-phosphate dehydrogenase	NM_000402	GATGTCCCCTGTCCCACCAACTCTG/GCAGGGCATTGAGGTTGGGAG

### Western blot analysis

Cultures were harvested in lysis buffer containing protease inhibitors. For immunoblotting, 12 μg of protein homogenate diluted in sodium dodecyl sulfate (SDS) buffer was heated at 37°C for 3 minutes and separated on a 10% SDS–polyacrylamide gel. Proteins were transferred to nitrocellulose membranes (1 hour at 100 V), and unspecific sites were blocked with 5% nonfat milk diluted in wash solution for 1 hour at room temperature. Membranes were then incubated overnight at 4°C with the primary antibody against AKR1B1 (dilution 1/1000, provided by Dr Fortier [45]), AKR1C3 (dilution 1/1000, Acris Antibody Inc., Sandiego, CA, USA), COX-2 (dilution 1/3000, provided by Dr S Kargman (Merck, Quebec, Canada)) or β-tubulin (Cell Signaling Technology, Danvers, MA, USA) as loading control, washed 4 × 15 minutes, and incubated for 1 hour with anti-rabbit immunoglobulin G conjugated to horseradish peroxidase. Finally, membranes were washed 4 × 15 minutes and proteins were visualized by chemiluminescence. Densitometric analysis of protein levels was performed using Image J software (NIH, USA).

### Immunohistochemistry

Adipose tissue macrophage infiltration was quantified by fluorescence immunostaining on formalin-fixed and paraffin-embedded adipose tissue samples, as previously described [1]. Immunostaining for CD68 (mouse anti-human CD68 antibody, Cedarlane, Burlington, Ontario, Canada) was performed and the number of cells infiltrated by macrophages was counted (identified as CD68+ cells) in a blinded manner. A minimum of 400 adipocytes were examined for each sample. The number of macrophages was normalized for 100 adipocytes.

### Statistical analyses

Repeated-measures analysis of variance in each fat compartment was used to compare mean PGF_2α_ release by preadipocytes, isolated mature adipocytes or adipose tissue explants in response to TNF-α and/or IL-1β and in the presence or absence of increasing concentrations (0–20 μM) of ponalrestat. Repeated-measures analysis of variance was also performed to compare mean COX-2, ARK1B1 and AKR1C3 expression levels by preadipocytes in response to TNF-α and/or IL-1β. Pearson correlation coefficients were computed to quantify associations between adipose tissue mRNA expression of *AKR1B1* and adiposity as well as metabolic measurements. PGF_2α_ release by mature adipocytes was high in approximately a third of the patients (n = 12) in each fat compartment and relatively low in the remaining patients. For this reason, PGF_2α_ release by mature cells was analyzed in categorical fashion. Indeed, women were subdivided in two subgroups with either low (n = 23) or high (n = 12) PGF_2α_ release in each fat compartment. Patients with undetectable PGF_2α_ release by mature adipocytes (n = 5 patients for both compartments and in n = 2 patients in one of the compartments) were included in the group characterized with low PGF_2α_ release. Adiposity measurements and metabolic outcomes were compared in women with low vs. high PGF_2α_ release using Student's t tests. All data were presented as mean ± SEM. Log_10_ and box-cox transformations were used for non-normally distributed variables. Statistical analyses were performed with the JMP 4.0 software (SAS Institute, Cary, NC).

## Results

### PGF_2α_ release by primary preadipocytes, mature adipocytes and adipose tissue explants from the subcutaneous and omental compartments

**Figure 1:**
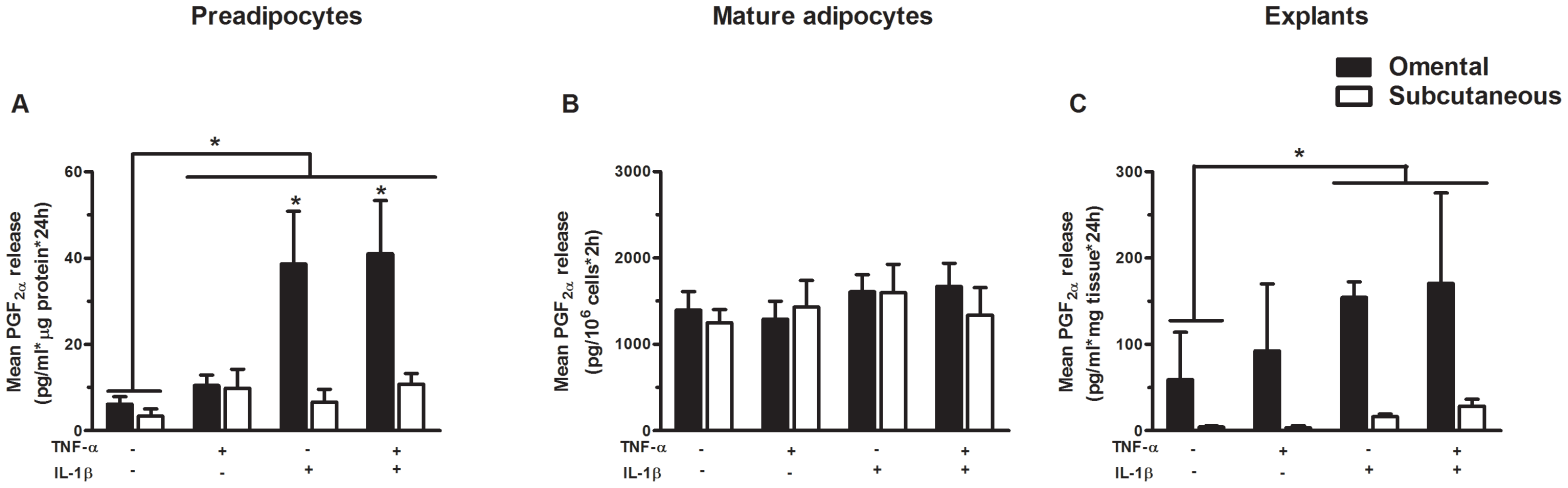
PGF_2α_ release by omental and subcutaneous fat cells. (**A**) PGF_2α_ release by subcutaneous and omental primary preadipocytes in response to TNF-α and/or IL-1β (preadipocytes stimulated for 24 h with 1 ng/ml TNF-α, 1 ng/ml IL-1β or both). Results are expressed as pg/ml*μg protein*24 h (n = 14), (**B**) PGF_2α_ release by isolated subcutaneous and omental mature adipocytes in response to TNF-α and/or IL-1β (isolated mature adipocytes stimulated for 2 h with 1 ng/ml TNF-α, 1 ng/ml IL-1β or both). Results are expressed as pg/10^6^cells*2 h (n = 12), (**C**) PGF_2α_ release by subcutaneous and omental adipose tissue explants in response to TNF-α and/or IL-1β (explants stimulated for 24 h with 1 ng/ml TNF-α, 1 ng/ml IL-1β or both). Results are expressed as pg/ml*mg tissue*24 h. Data are presented as mean ± SEM. p≤0.05 for treatment-by-depot interaction in panel A and p≤0.05 for treatment effect in panels A and C. * p ≤ 0.05.

**Figure 1** shows PGF_2α_ release by subcutaneous and omental preadipocyte primary cultures over 24 hours, collagenase-isolated mature adipocyte suspensions over 2 hours or explants over 24 hours. **Figures 1A** shows the release of PGF_2α_ by subcutaneous and omental primary preadipocyte cultures following stimulation with TNF-α and/or IL-1β. PGF_2α_ release was significantly induced in response to TNF-α and/or IL-1β compared to control in omental (p = 0.01) and to a lesser extent in subcutaneous preadipocytes (p = 0.02). PGF_2α_ release by omental preadipocytes was significantly higher in response to IL-1β and combined TNF-α and IL-1β compared to that of subcutaneous preadipocytes, with a significant treatment-by-depot interaction (p≤0.05).

Using repeated-measures analysis of variance, PGF_2α_ release was not significantly increased in response to short-term TNF-α and/or IL-1β treatments compared to control in omental or subcutaneous mature adipocytes. No significant depot difference was observed in PGF_2α_ release by omental and subcutaneous mature adipocytes in all conditions tested suggesting that the effect of IL-1β and TNF-α may be transcriptional and may require longer incubation times (**Figure 1B**).

Similar to preadipocyte cultures, **Figures 1C** shows that PGF_2α_ release was significantly induced in response to IL-1β or combined TNF-α and IL-1β compared to control in explants from both fat compartments (p = 0.01). Omental explants tended to have a higher PGF_2α_ release in response to TNF-α and IL-1β compared to subcutaneous explants, but this difference did not reach significance.

### COX-2 expression and PGF synthase expression in primary preadipocytes

**Figure 2:**
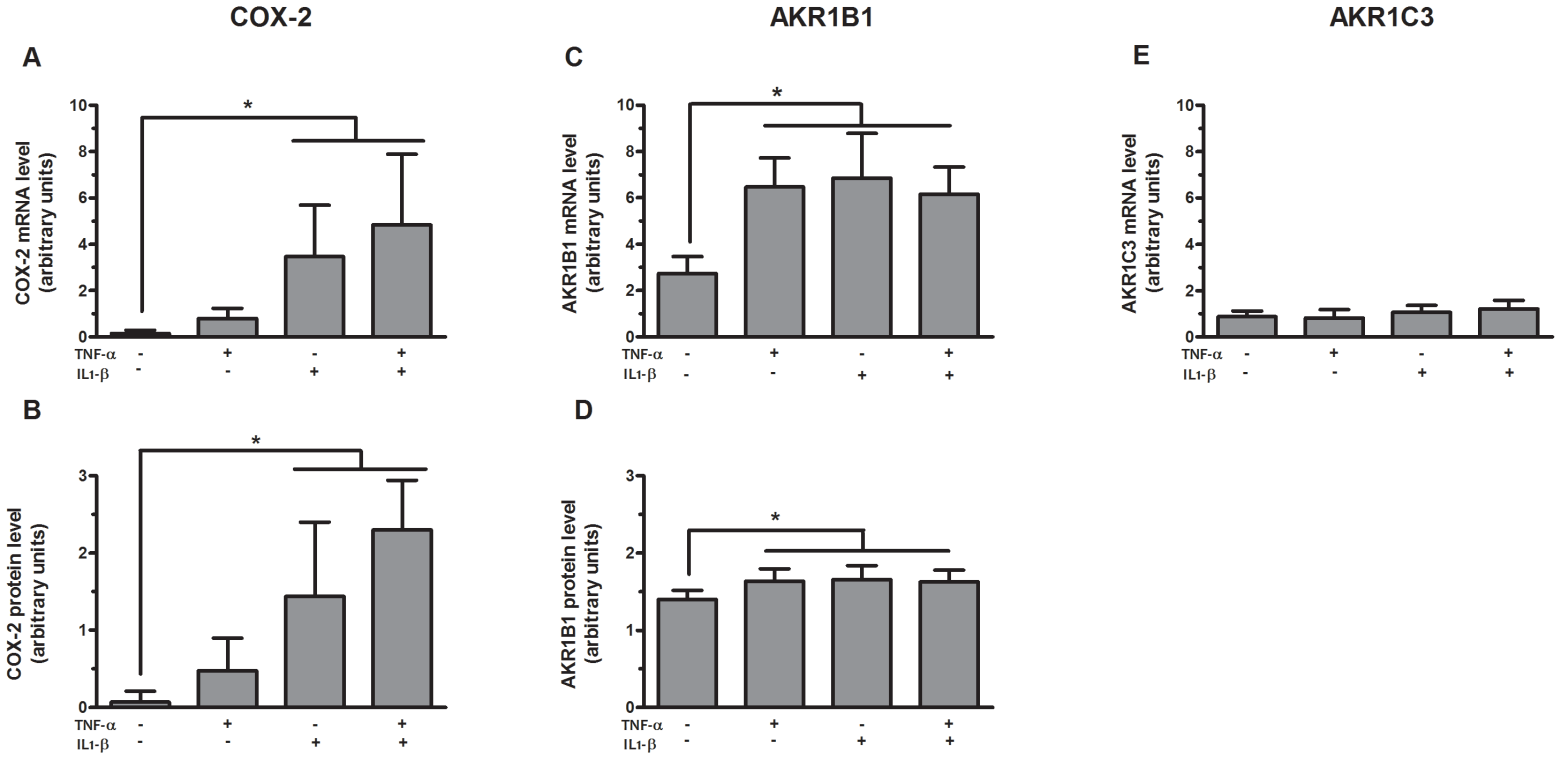
COX-2 and PGF synthase expression in primary preadipocytes. Messenger RNA expression and protein levels of COX-2 (**A** and **B**, respectively), mRNA expression and protein levels of AKR1B1 (**C** and **D**, respectively) and mRNA expression of *AKR1C3* (**E**) in subcutaneous and omental preadipocytes (n = 4) stimulated for 24 h with 1 ng/ml TNF-α, 1 ng/ml IL-1β or both. The data are presented as mean ± SEM (* p≤0.05 for treatment effect in panels A, B, C and D). Expression levels relative to G6PD mRNA expression. The western blot data were quantified by densitometric analysis and values were normalized to β-tubulin.

**Figure 3:**
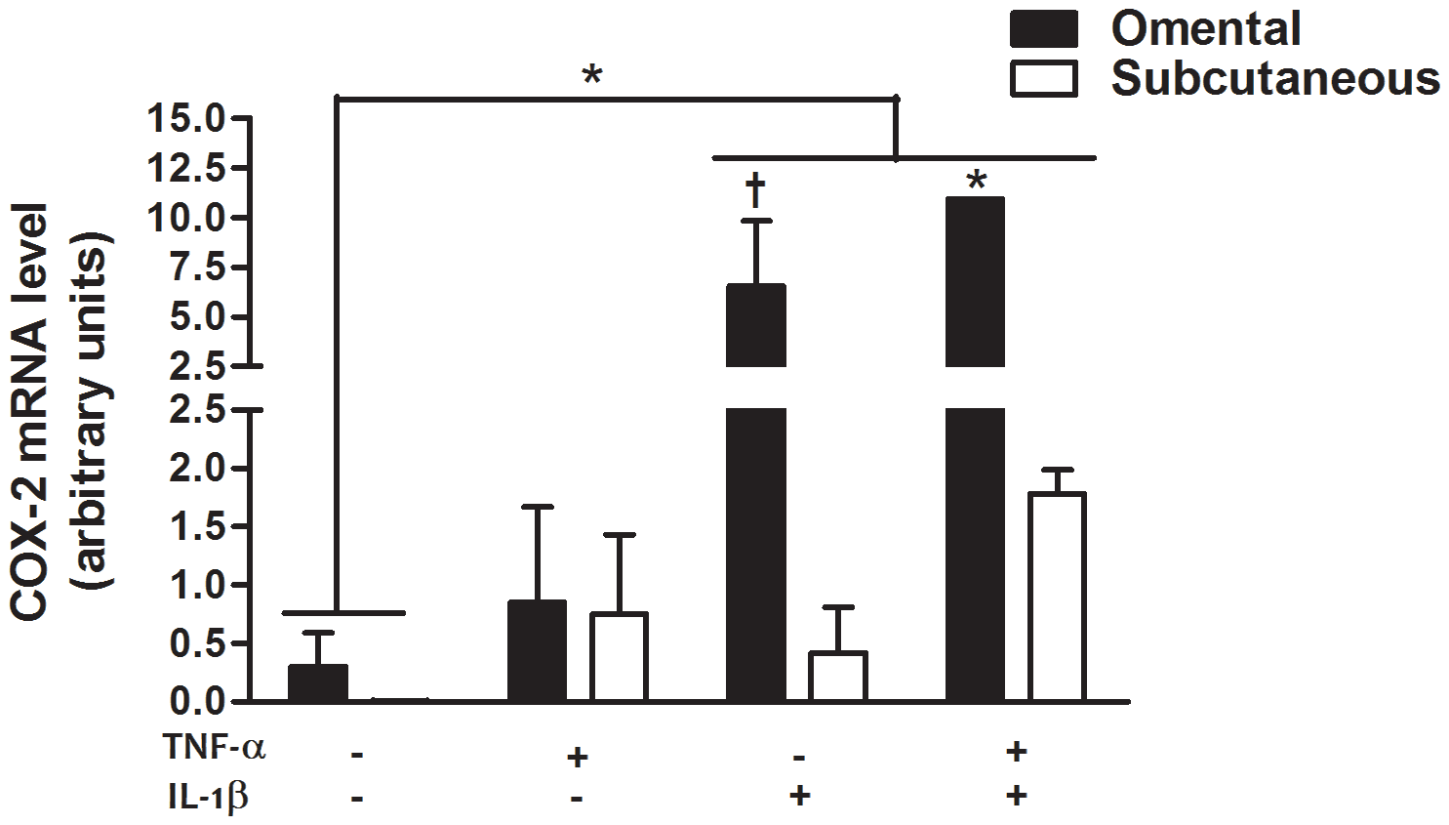
Depot differences in COX-2 expression in primary preadipocytes. Messenger RNA expression of COX-2 in subcutaneous (n = 2) and omental (n = 2) preadipocytes stimulated for 24 h with 1 ng/ml TNF-α, 1 ng/ml IL-1β or both. Data are presented as mean ± SEM * p≤0.05, † p≤0.08. Expression levels relative to G6PD mRNA expression.

Considering that inflammatory cytokines significantly induced PGF_2α_ release by cultured preadipocytes (**Figure 1**), we also examined the expression of COX-2 and the two putative terminal PGF synthases, AKR1B1 and AKR1C3, in cultured preadipocytes in response to inflammatory cytokines (**Figure 2**). Messenger RNA expression (**Figure 2A**) and protein levels (**Figure 2B**) of COX-2 in cultured preadipocytes were significantly increased in response to IL-1β and combined TNF-α and IL-1β compared to control (p≤0.05). Interestingly, abundance of *COX-2* mRNA was significantly higher in omental compared to subcutaneous preadipocytes in response to combined TNF-α and IL-1β (p = 0.01) (**Figure 3**). No significant depot difference was observed in protein levels of COX-2 (data not shown). Messenger RNA expression (**Figure 2C**) and protein levels (**Figure 2D**) of AKR1B1 in cultured preadipocytes were also significantly increased in response to TNF-α and/or IL-1β compared to control (p≤0.05). No significant depot difference was observed in mRNA expression and protein levels of AKR1B1 (data not shown). Interestingly, TNF-α and/or IL-1β treatment in cultured preadipocytes failed to increase expression of the other putative terminal PGF synthase *AKR1C3* and although subcutaneous mRNA expression of this gene tended to be higher compared to omental cultured preadipocytes, this trend did not reach significance (**Figure 2E**). Furthermore, even if we detected the recombinant protein of AKR1C3 (positive control), protein levels of AKR1C3 were not detected in cultured preadipocytes (data not shown). Similar to results of **Figure 1**, PGF_2α_ release was also significantly induced in response to TNF-α and/or IL-1β compared to control in these cultures (p = 0.02) (data not shown).

### Effect of aldose reductase inhibitor on PGF_2α_ release by human primary preadipocytes

**Figure 4:**
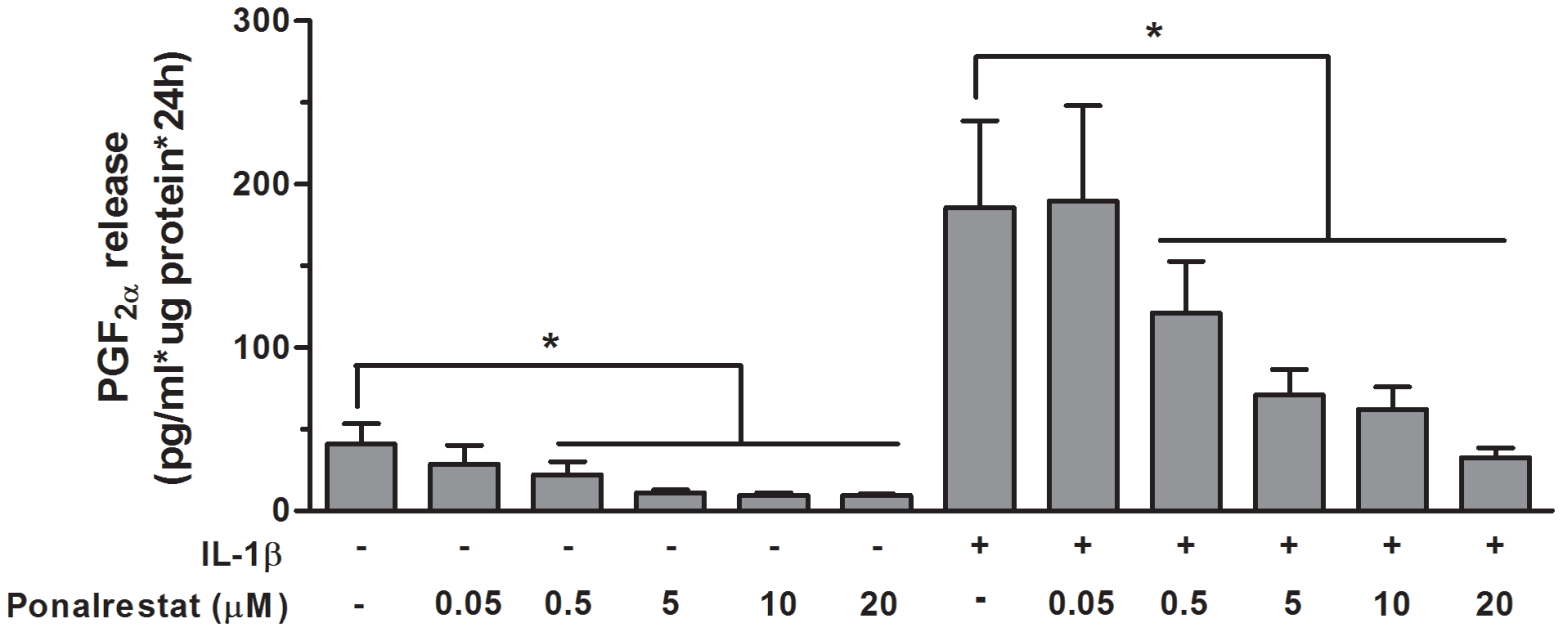
Effect of aldose reductase inhibitor on PGF_2α_ release by human primary preadipocytes. PGF_2α_ release by subcutaneous and omental preadipocytes treated for 24 h with 1 ng/ml IL-1β in the presence or absence of increasing concentrations (0-20 μM) of ponalrestat. Data are presented as mean ± SEM. Results are expressed as pg/ml*μg protein*24 h (* p≤0.05, n = 7 for all conditions).

Considering that inflammatory cytokines increased the expression of AKR1B1 at the mRNA and protein levels, but failed to increase the expression of AKR1C3, and that protein levels of AKR1C3 were not detected in preadipocytes, we examined the effect of ponalrestat, an aldose reductase inhibitor developed to inhibit the conversion of glucose to sorbitol by AKR1B1, on PGF_2α_ release by human primary preadipocytes. **Figure 4** illustrates that ponalrestat completely reversed the stimulatory effect of IL-1β on PGF_2α_ release by human primary preadipocytes, in a dose-dependant manner (p≤0.05). Pronalrestat also significantly inhibited the production of PGF_2α_ by cultured preadipocytes in basal conditions (p≤0.05). Ponalrestat had no effect on cell viability.

### Expression levels of AKR1B1 in whole subcutaneous vs. omental adipose tissue

Considering that AKR1B1 seems to have a predominant role in PGF_2α_ synthesis by human preadipocytes in response to inflammatory cytokines, we measured whole subcutaneous and omental adipose tissue expression of *AKR1B1* in a sample of 46 women exhibiting lean to severely obese phenotypes. **Table 2** summarizes the characteristics of the study sample. Messenger RNA levels of *AKR1B1* were detectable in tissues from both fat compartments and were higher in omental compared to subcutaneous adipose tissue (p≤0.01, data not shown). Positive and significant correlations were observed between whole tissue *AKR1B1* mRNA expression in both compartments, and BMI, waist circumference as well as HOMA_ir_ index (p≤0.05, for all) (**Table 3**).

**Table 2:** **Characteristics of the sample (n = 46).**

Variables	Mean ± SD			Range (min-max)
Age (yrs)	46.8 ± 4.0			37.6–54.5
Body weight (kg)	74 ± 17			48–133
Waist circumference (cm)^a^	94 ± 15			72–147
BMI (kg/m^2^)	28.0 ± 6.4			19.5–50.1

a n = 45.

**Table 3:** **Pearson correlation coefficients between** ***AKR1B1*** **mRNA expression level in subcutaneous (SC) or omental (OM) adipose tissue and anthropometric measurements or HOMA_ir_ Index (n = 46).**

Variables	*AKR1B1*	
	OM	SC
BMI	0.30*	0.46**
Waist circumference^a^	0.29^†^	0.51**
HOMA_ir_ Index	0.34*	0.58**

*AKR1B1* mRNA expression in whole tissue from each site. Expression levels relative to *ATP5O* mRNA expression, ^a^n = 45, ** p≤0.005, *p ≤ 0.05, ^†^p ≤ 0.10.

### PGF_2α_ release by subcutaneous and omental mature adipocytes in relation with body fatness, glucose homeostasis and adipose tissue macrophage infiltration

**Figure 5:**
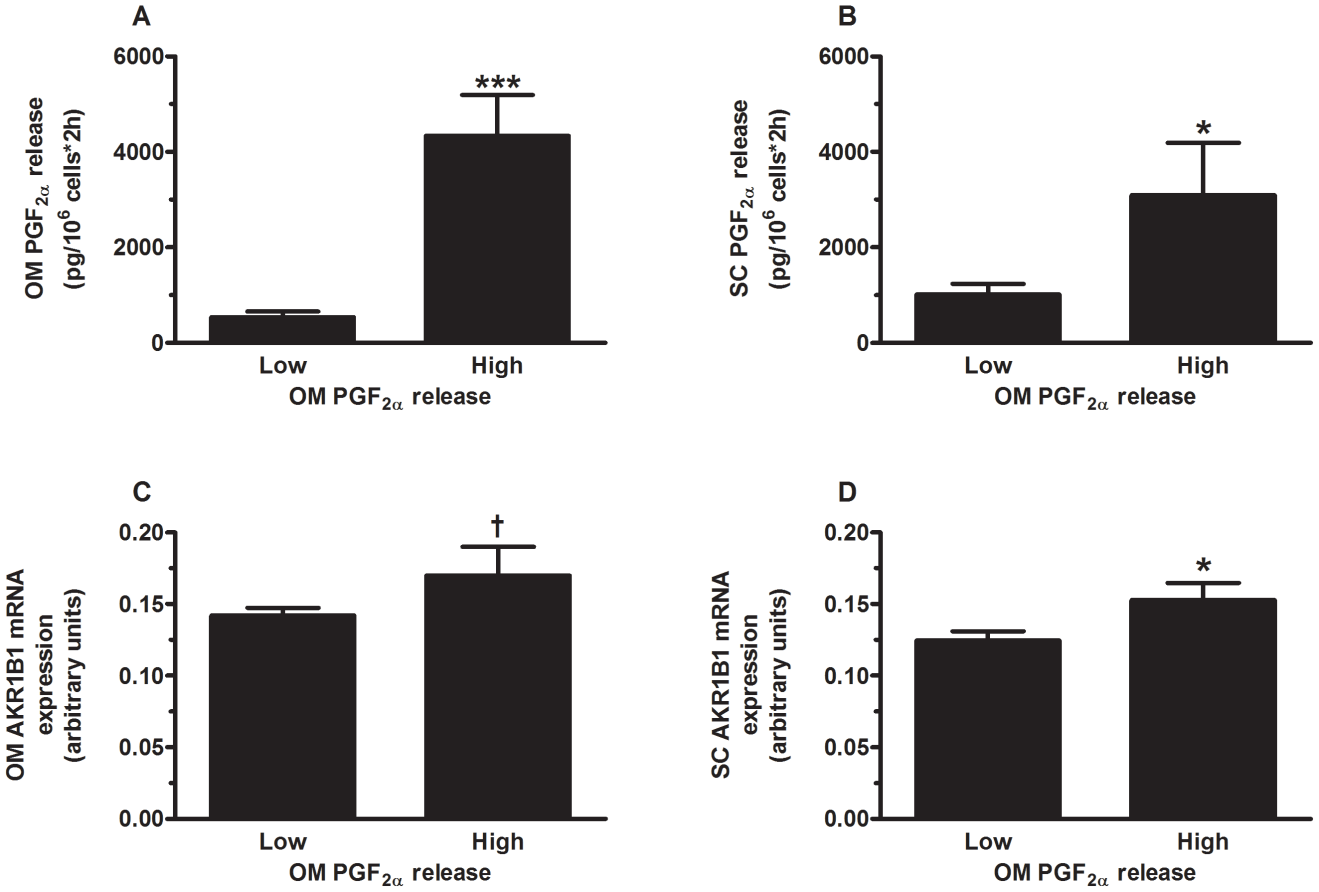
PGF_2α_ release by omental mature adipocytes. Comparison of (**A**) omental adipocyte PGF_2α_ release; (**B**) subcutaneous adipocyte PGF_2α_ release; (**C**) omental *AKR1B1* mRNA expression; and (**D**) subcutaneous *AKR1B1* mRNA expression in women with low or high omental adipocyte PGF_2α_ release. Data are presented as mean ± SEM. ^†^ p < 0.10, *p ≤ 0.05, **p ≤ 0.005, *** p ≤ 0.0001. Expression levels relative to *ATP5O* mRNA expression. OM: omental; SC: Subcutaneous.

PGF_2α_ release by subcutaneous and omental mature adipocyte suspensions was measured in a subsample of the study (35 women exhibiting lean to severely obese phenotypes) for which we have prepared isolated mature adipocytes from the omental and subcutaneous fat compartments. Women were subdivided in two subgroups with either low (n = 23) or high (n = 12) omental adipocyte PGF_2α_ release. According to this stratification, women with the highest PGF_2α_ release by omental adipocytes had significantly higher omental adipocyte PGF_2α_ release compared to women with low omental adipocyte PGF_2α_ release (p≤0.0001) (**Figure 5A**). Women with the highest PGF_2α_ release by omental adipocytes also had significantly higher PGF_2α_ release by subcutaneous adipocytes (p≤0.05) (**Figure 5B**) compared to women with low PGF_2α_ release by omental adipocytes. These women also tended to have higher omental adipose tissue mRNA expression of *AKR1B1* (p<0.10) and had significantly higher subcutaneous adipose tissue mRNA expression of *AKR1B1* (p≤0.05) (**Figure 5C and D**).

**Figure 6:**
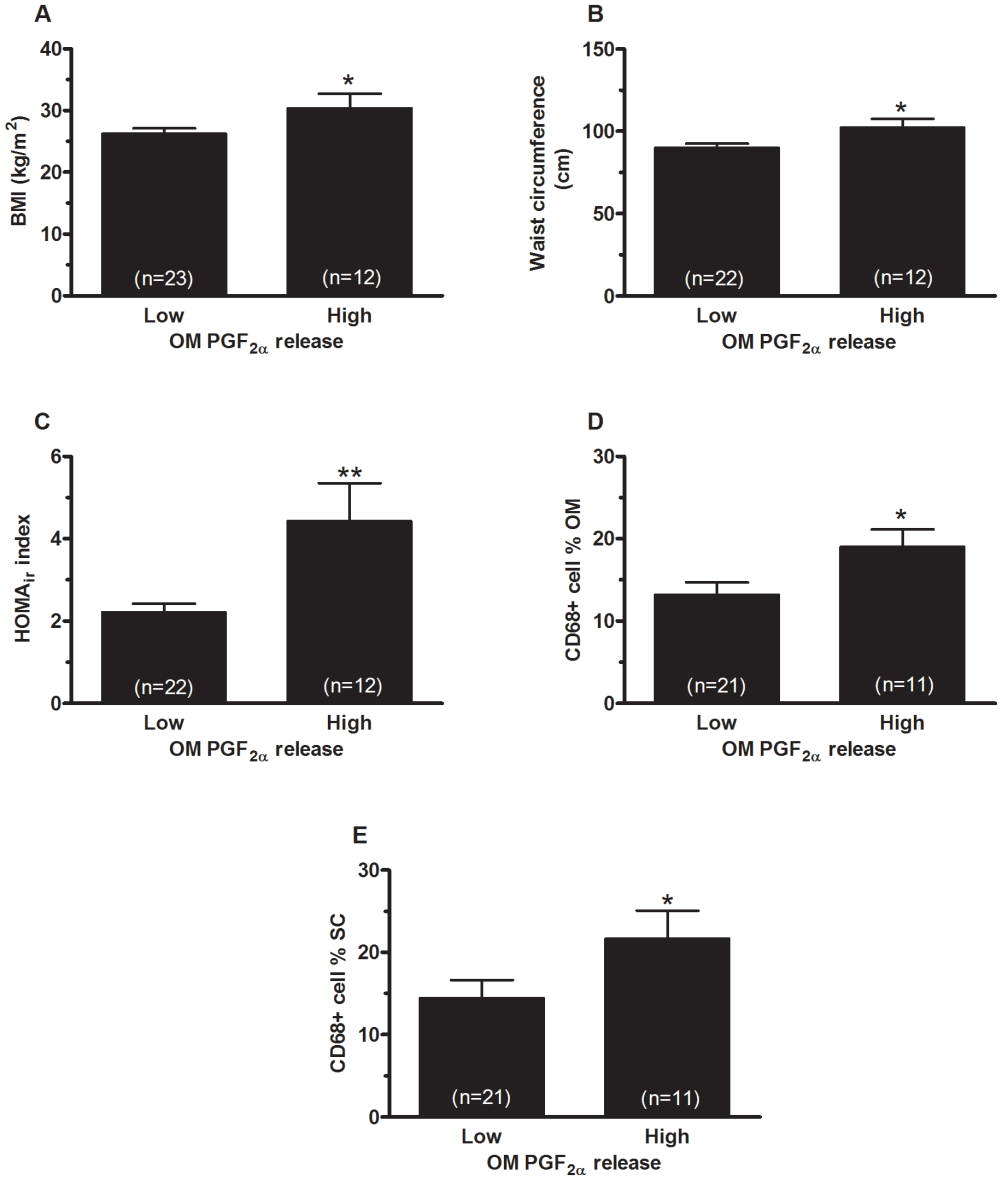
PGF_2α_ release by omental mature adipocytes in relation with body fatness and metabolic variables. Comparison of (**A**) BMI; (**B**) waist circumference; (**C**) HOMA_ir_ index; (**D**) omental and (**E**) subcutaneous adipose tissue CD68+ cell percentage in women with low or high omental adipocyte PGF_2α_ release. Data are presented as mean ± SEM. *p ≤ 0.05, ** p ≤ 0.005.

Women with the highest PGF_2α_ release by omental adipocytes had significantly higher BMI (p = 0.05) (**Figure 6A**), waist circumference (p≤0.05) (**Figure 6B**) and omental adipocyte diameter (p≤0.005) (data not shown) compared to women with low omental PGF_2α_ release. Regarding glucose homeostasis, women with the highest PGF_2α_ release by omental adipocytes had significantly higher fasting glycemia (p≤0.005) (data not shown), fasting insulinemia (p≤0.05) (data not shown) and HOMA_ir_ index (p≤0.005) (**Figure 6C**) compared to women with low omental adipocytes PGF_2α_ release. Finally, regarding adipose tissue macrophage infiltration, women with the highest omental PGF_2α_ release had significantly more CD68+cells in both omental (p≤0.05) (**Figure 6D**) and subcutaneous adipose tissue (p≤0.05) (**Figure 6E**) compared to women with low PGF_2α_ release. PGF_2α_ release by subcutaneous mature adipocytes was not significantly related to metabolic parameters. Only statistical trends were observed.

## Discussion

To our knowledge, this is the first study to clearly examine fat depot-specific differences in PGF_2α_ release by human subcutaneous and omental adipose tissue cell fractions. We found that PGF_2α_ release was significantly induced in response to TNF-α and/or IL-1β compared to control in omental and to a lesser extent in subcutaneous preadipocytes. Higher mRNA expression levels of COX-2 were observed in omental compared to subcutaneous stimulated preadipocytes, suggesting that the change in COX-2 expression in response to inflammatory cytokines is an important regulator of PGF_2α_ production in preadipocytes. We also observed that inflammatory cytokines stimulated AKR1B1 mRNA and protein levels, but failed to increase expression levels of *AKR1C3* in preadipocytes, suggesting that AKR1B1 is a likely candidate for cytokine-stimulated PGF_2α_ synthesis in preadipocytes, as opposed to AKR1C3. Accordingly, our experiment with ponalrestat demonstrated that AKR1B1 may have a predominant role in the production of PGF_2α_ synthesis by cultured preadipocytes in response to basal and inflammatory conditions. We also found that PGF_2α_ release by omental mature adipocytes and whole tissue mRNA expression of *AKR1B1* is increased in abdominally obese women with altered glucose homeostasis.

One important finding in this study is that AKR1B1 has a predominant role in PGF_2α_ synthesis by human preadipocytes in response to inflammatory cytokines compared to AKR1C3. AKR1C3 is known to exhibit ketosteroid reductase activity (type 5 17β-HSD), which mostly inactivates progesterone into 20α-hydroxyprogesterone [46]. AKR1C3 also displays 17β-HSD and 3α-HSD activities [46], [47]. Theses studies demonstrated that AKR1C3 is highly expressed in subcutaneous adipose tissue and seems to have steroid reductase activities in human abdominal adipose tissue samples [48]. In the present study, we observed that cytokine treatment stimulated PGF_2α_ release as well as the mRNA and protein levels of AKR1B1 and COX-2, but failed to increase expression levels of AKR1C3 in preadipocytes. Furthermore, AKR1C3 protein levels were not detected in cultured preadipocytes treated with cytokines or vehicle while AKR1C3 mRNA expression levels were very low, further excluding a potential role of AKR1C3 in PGF_2α_ synthesis by preadipocytes. We also found that ponalrestat, an inhibitor of AKR1B1, significantly decreased the inflammatory effect of IL-1β on PGF_2α_ production by primary preadipocytes. These results further suggest that AKR1BI may be relevant for PGF synthase in human preadipocytes. Consistent with our results, Kabututu et al. demonstrated that recombinant AKR1B1, Akr1b3 and Akr1b7 had better PGF synthase activities than previously-characterized PGF synthases (AKR1C family members) in mammals [36]. We also previously showed that AKR from the 1B family exhibited PGF synthase activity, first in the bovine model [33], but also in human endometrium [34] and other tissues [35], which is consistent with the present results. Indeed, we previously demonstrated that the reduction of AKR1B1 by specific siRNA knockdown was related to a significant decrease in PGF_2α_ release [34]. The PGF synthase activity of AKR1B1 has also been established by others [49]–[51]. Fujimori et al. also demonstrated that siRNA for Akr1b3 suppresses PGF_2α_ synthesis in the 3T3-L1 cell line, indicating that Akr1b3 is the primary PGF synthase in mouse preadipocytes [37]. Consistent with these studies, we establish for the first time in human adipose tissue that preadipocyte release of PGF_2α_ is responsive to inflammatory stimulation and that AKR1B1 may be largely responsible for this response.

We demonstrated that preadipocytes from the omental fat compartment released more PGF_2α_ in response to inflammatory stimuli compared to those from subcutaneous fat. The higher mRNA expression of COX-2 in omental compared to subcutaneous preadipocytes after inflammatory stimulation very likely explains these depot differences. Indeed, we found no significant depot differences in the expression of AKR1B1 in stimulated preadipocytes. Consistent with our results, previous studies had demonstrated that COX-2 is induced by inflammatory stimulation and is the rate-limiting enzyme in the synthesis of PG [51]. Even if COX-2 explains depot differences and is implicated in PGF_2α_ production, our experiment with ponalrestat demonstrated that blockade of AKR1B1 still inhibits most of the PGF_2α_ synthesis by cultured preadipocytes either in the basal state or in response to inflammatory stimulation. Our results indirectly suggest a predisposition of omental fat cells to respond to inflammation through this mechanism. The particular sensitivity of preadipocytes from the omental fat depot in response to inflammatory stimulation possibly plays a pathophysiological role in visceral obesity.

We also demonstrated that women with elevated omental adipocyte PGF_2α_ release have a significantly higher BMI and waist circumference. Consistently, obesity level was significantly and positively related to adipose tissue mRNA expression levels of *AKR1B1*. The physiological consequences of these observations remain unclear. McQuaid et al. recently demonstrated that abdominally obese subjects had significantly lower adipose tissue blood flow in the fasting and postprandial states compared to lean subjects [2]. More specifically, they demonstrated that abdominal obesity is associated to adipose tissue adaptation in terms of systemic non-esterified fatty acid (NEFA) delivery and vascular functions of the tissue, which seem to be involved in fat storage dysfunction and ectopic fat deposition [2]. Farb et al. also recently demonstrated that cyclooxygenase-derived vasoconstrictor prostanoids may contribute to endothelial dysfunction of visceral adipose arterioles [52]. Considering that PGF_2α_ is an important vasoconstrictor [31], high release of PGF_2α_ by adipose cells of women with abdominal obesity may contribute to these phenomena and may represent an indicator of adipose tissue dysfunction. We also observed that women with elevated omental adipocyte PGF_2α_ release have increased adipose tissue macrophage infiltration. In addition, mature omental adipocytes that released the highest amounts of PGF_2α_ were those with the largest size, indirectly suggesting that omental adipocyte hypertrophy may be a determinant of this secretory function of the cell. Accordingly, other studies have demonstrated that adipocyte hypertrophy creating local hypoxic conditions may be involved in the attraction of macrophages by stimulating inflammatory pathways such as JNK1-regulated chemokine release [5], [53], [54]. The elevated release of PGF_2α_ by mature fat cells of women with abdominal obesity may either reflect or contribute to these phenomena as a mediator of inflammation.

The main PG-secreting cells are in the stromal-vascular fraction of adipose tissue [10], [11], [15]. In the present study, we could neither confirm nor contradict this notion due to methodological differences in culture conditions. We used mature fat cell suspensions normalized for the amount of cells on the one hand, and adherent preadipocytes in primary cultures on the other, with data expressed as a function of protein level. The reasons for this methodological discrepancy relates to the nature and cultivability of each cell type. Mature fat cells cannot be kept more than a few hours in suspensions. Conversely, we could not study prostaglandin release in freshly isolated stromal-vascular fractions due to the small cell numbers present in our samples. These methodological limitations in the culture models may explain why the depot difference in PGF_2α_ release was not significant in some of the conditions tested. Short incubation times (2 hours) of the mature adipocyte preparations may explain the lack of stimulation by inflammatory cytokines. Furthermore, the lack of a significant depot difference in the release of PGF_2α_ by adipose tissue explants may relate to the small number of cultures. We also failed to observe depot difference in COX-2 protein levels in our samples, but we suggest that this discrepancy may be related to the smaller number of samples examined in western blot experiments. Quantitative realtime PCR measurements are generally more sensitive to assess fat depot differences with relatively small sample sizes compared to protein measurements. We also observed that PGF_2α_ release by subcutaneous mature adipocytes was not significantly related to all metabolic parameters. We suggested that the wider range of variability in visceral adipose tissue area and omental adipocyte size in that sample favored stronger correlations of a given parameter measured in visceral fat compared to the same parameter in subcutaneous adipose tissue. Furthermore, power analysis suggests that a much larger sample size would have been required to observe significant differences in metabolic parameters between women with either low or high PGF_2α_ release by subcutaneous adipocytes. In spite of these limitations, we show that omental preadipocytes have a higher capacity to release PGF_2α_ and respond to inflammatory stimuli compared to subcutaneous preadipocytes. Another acknowledged limitation of the study is the absence of male subjects. The main reason for not including men is the difficulty to obtain comparable samples from generally healthy lean to moderately obese subjects. We also assessed total macrophage infiltration using CD68 as the sole marker. Other markers of macrophage infiltration may have generated different results.

In conclusion, we found that omental preadipocyte release of PGF_2α_ is particularly responsive to inflammatory stimulation. Higher expression levels of *COX-2* observed in omental compared to subcutaneous stimulated preadipocytes suggests that the changes in COX-2 expression are an important regulator of PGF_2α_ production in preadipocytes. This study also demonstrates for the first time that AKR1B1 may have a predominant role in PGF_2α_ synthesis by human preadipocytes in response to inflammatory cytokines compared to AKR1C3. In this context, further studies are needed to examine the role of human AKR1B1 as a PGF synthase in various cells of adipose tissue and how it might modulate adipose tissue homeostasis in humans.
